# Reintroduced White Storks ( *Ciconia ciconia* ) Have Similar Diets to Their Wild Conspecifics

**DOI:** 10.1002/ece3.71278

**Published:** 2025-04-11

**Authors:** Şeniz Mustafa, Connor T. Panter, Laura Vaughan‐Hirsch, Rachel L. White, Anja Rott

**Affiliations:** ^1^ School of Applied Sciences University of Brighton Brighton UK; ^2^ School of Geography University of Nottingham Nottingham UK; ^3^ White Stork Project Knepp Castle Estate Horsham UK

**Keywords:** avian diets, conservation interventions, conservation translocations, human‐nature interactions, rewilding, trophic ecology

## Abstract

Understanding a species' diet is crucial for assessing its ecology and can indicate the success of reintroduction efforts. We explored dietary composition and compared pellet morphology and supplementary prey proportions between two white stork ( *Ciconia ciconia* ) groups to assess reintroduction effectiveness. White stork groups consisted of released individuals that were free flying (i.e., “wild group”) and those kept within a confined enclosure (“captive group”). A total of 23 white stork pellets were collected during the 2023 breeding season. Wild group pellets were significantly heavier ($\bar{x}$ = 12.7 ± 9.2 g [SD]) than captive group pellets ($\bar{x}$ = 5.2 ± 2.1 g). As expected, all captive group pellets contained supplementary prey, accounting for 88.4% ± 26.1% of pellet biomass, whereas 73.3% of wild group pellets contained supplementary prey, comprising 52.9% ± 36.3% of pellet biomass. The wild group predominantly foraged on beetles (Coleoptera) and earthworms (Clitellata). Our results represent the first quantitative dietary assessment of reintroduced white storks in southern England. Similarities between our data and that of wild white stork diets from elsewhere in their range suggest successful post‐release acclimatisation at Knepp Estate.

## Graphical abstract

We explored the dietary composition and compared pellet morphology using pellet analysis (*n* = 23) between two White Stork ( *Ciconia ciconia* ) groups to assess reintroduction effectiveness that consisted of released individuals that were free flying (i.e., “wild group”) and those kept within a confined enclosure (“captive group”). We found that 60% of the pellets from wild groups contained supplementary prey, making up 53% of the total biomass, and predominantly foraged on beetles (Coleoptera) and earthworms (Clitellata). Similarities between our data and wild White Stork diets from elsewhere in their range suggest successful post‐release acclimatisation at Knepp Estate.

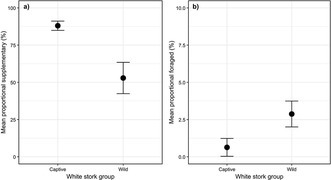

## 1 Introduction

The overarching aim of most reintroductions is to establish a viable and self‐sustaining population (Hayward et al. 2019; Miller et al. 2014; Zhang et al. 2021) either for (i) species conservation (Ottewell et al. 2014; Moraes et al. 2017), where the goal is to re‐establish a viable population to improve its status locally or globally (Houde et al. 2015; Batson et al. 2015; Seddon et al. 2014; Swan et al. 2016) or (ii) as part of wider initiatives aiming to restore natural ecosystem functions or processes (Zamboni et al. 2017; Houde et al. 2015). For example, in 1980, the red kite ( *Milvus milvus* ) was successfully reintroduced to the Chilterns in England and the Black Isle in Scotland (Smart et al. 2010; Evans et al. 2022; Peniche et al. 2011; Carter and Grice 2000) with an estimated 3000 breeding pairs in England in 2017 (Molenaar et al. 2017). Post‐release monitoring is fundamental for any reintroduction initiative, and assessing changes in the diet of released individuals can act as a barometer of conservation translocation success (Muposhi et al. 2014) as dietary data can reveal how well a species acclimates to a release site (Bannister et al. 2021; Muposhi et al. 2014), how its role evolves as populations expand (Newsome et al. 2015), and whether conservation translocations are likely to be successful (Abram 2023).

The white stork (hereafter “stork”) ( *Ciconia ciconia* ) is a large bird in the family Ciconiidae. The species is classified as Least Concern on the International Union for the Conservation of Nature's Red List of Threatened Species (IUCN Red List) due to its large and increasing population size (BirdLife International 2024). The European population is estimated at 224,000–247,000 breeding pairs (BirdLife International 2024). It inhabits open landscapes, including inland wetlands (Hancock et al. 1992) and arable land (Snow and Perrins 1998). Storks breed in open nests, often on trees or rooftops (Tryjanowski et al. 2004; Vergara et al. 2010) and can live up to 25 years in the wild (Tryjanowski et al. 2004). They are a generalist species, mainly feeding on insects, earthworms, molluscs, small mammals, amphibians, reptiles, and small birds (Massemin‐Challet et al. 2006; Orłowski et al. 2016; Vrezec 2009).

Records of this species in the UK include remains ranging from over 4000 years old in Cornwall and 3000 years old on the Shetland Islands, then to more scattered finds dating from 1000 to 700 years ago (Schmölcke and Thomsen 2025). However, this presence does not confirm successful breeding nor whether these individuals were visitors or on migration. Storks have been absent in the UK for 660 years due to historical overhunting and wetland drainage (Mayall et al. 2023). A Southern attempt to reestablish a breeding population of storks at Knepp in West Sussex, England, was facilitated by the “White Stork Project” (WSP), which is a partnership of conservation organisations and private landowners (White et al. 2023). The project aims to produce a self‐sustaining population of 50 breeding pairs by 2030 (Mayall 2022) and improve the public's connection with nature (White et al. 2023). In 2016, a population of adult storks was released at Knepp Estate, comprising of flying and flightless individuals translocated from Poland (Knepp Castle Estate 2024a). Flightless storks were kept in an open enclosure for protection. In 2019, a closed quarantine enclosure was constructed for storks from the Cotswold Wildlife Park before their full release (Mayall 2022). Post‐release, flying storks foraged freely, often dispersing across southern England (Knepp Castle Estate 2024a). Successful breeding attempts occurred in 2020 and 2023, with offspring reared by both flying and non‐flying pairs (White Stork Project 2024).

Prior to and during the 2023 breeding season, storks were supplementarily fed with male chicks (*Gallus gallus domesticus*) and sprat ( *Sprattus sprattus* ). This soft‐release technique was used primarily to support the flightless storks due to their limited access to natural prey (Bannister et al. 2016; Cortés‐Avizanda et al. 2016; Fraga et al. 2023; Vaughan‐Hirsch 2024), and to improve the breeding success of the flying population by increasing clutch sizes and fledgling rates (Doerr et al. 2017; Ferreira et al. 2019; Galbraith et al. 2015; Maggs et al. 2019; Negro et al. 2007). However, this feeding is expected to end once the non‐flying individuals naturally reach the end of their lifespan (Vaughan‐Hirsch 2024). Understanding the impact of supplementary feeding is crucial for assessing the success of conservation translocations. For example, analysis of the Great Bustard ( *Otis tarda* ) diet revealed a high intake of supplementary prey during dispersal had impacted juveniles' ability to extract dietary nutrients and exhibit appropriate foraging behaviours (Gooch et al. 2015). Therefore, to assess the success of the stork reintroduction at Knepp Estate, it is essential to study the diet of storks to ascertain whether they rely on supplementary food post‐release, in addition to whether the surrounding habitats provide a suitable foraging diet and address concerns raised about the impact of stork predation on local species (White et al. 2023).

This study presents the first dietary assessment of reintroduced storks at Knepp Estate of flightless, that is, “captive group”, and free‐flying, that is, “wild group” storks. The captive group were flightless due to being quarantined in a covered pen. Both groups received supplementary food, but the wild group storks had access to additional foraging sites, allowing for greater prey variation to support their higher activity levels from breeding and flying (Nagy 2001; Turko et al. 2023). Because of this, we expect wild group pellets to be larger, heavier, and contain more foraged prey compared to the captive group. Due to restrictions imposed by the captive group enclosure, we expect supplementary prey to constitute the majority of the captive group pellet biomass. Based on other studies (see Orłowski et al. 2016, 2018; Surdo et al. 2022; Vrezec 2009), we anticipate that wild group pellets will contain a higher proportion of foraged prey, particularly Orthoptera and Coleoptera, due to their preference and availability in foraging habitats.

## 2 Materials & Methods

### 2.1 Study Area

The study took place in the southern block of Knepp Estate (50.971668, −0.362584), which is a 1400‐ha estate (Dempsey 2021; Tree 2017) of heavy weald clay (Bühne et al. 2022). Knepp Estate was previously farmed intensively until the early 2000s (Wallace 2018) and has since been managed as a rewilding site (Knepp Castle Estate 2024b). Large herbivores have been reintroduced into the site to restore lost ecological functions, including English Longhorn Cattle ( *Bos taurus* ), Tamworth Pigs ( *Sus scrofa domesticus* ), Exmoor Ponies ( *Equus ferus caballus* ), European Fallow Deer ( *Dama dama* ) and Red Deer ( *Cervus elaphus* ). The site is divided into “blocks”, with the southern block dominated by substantial scrub coverage (particularly sallow trees; *Salix* spp.), tall herb vegetation (Ryland 2015), grasslands (English Nature 2005) and wetlands fed by the River Adur tributaries (King 2016).

### 2.2 Pellet Collection and Analysis

From June till September 2023, pellets were collected from two groups of storks: a “captive group” bred at Cotswold Wildlife Park, which were confined to an enclosure for quarantining, and a “wild group” free‐flying with access to natural habitats (June to September 2023 with help from volunteers and White Stork Project staff). Captive group pellets were collected by hand from the enclosure, while wild group pellets were collected underneath active nests in English Oak ( *Quercus robur* ) trees outside the enclosure. Pellets that appeared clearly fragmented were excluded from this study. Both groups were offered supplementary food (chicken and sprat). Collected pellets were carefully labelled with the date and location, stored in sealed bags, and frozen at −18°C on‐site before being moved to the University of Brighton laboratories to prevent degradation. The number of pellets used in this study was restricted due to the time constraints associated with the academic course for which it was conducted.

To compare pellet morphology between the wild and captive groups, we measured pellet length (cm), width (cm), weight (g), and prey weights to the nearest 0.01 g. Pellets were soaked and washed through 1, 0.5, and 0.25 mm sieves to soften them for dissection, filter out larger specimens, and obtain polychaete and oligochaete remains (Antczak et al. 2002; Battisti et al. 2019). Chaete presence was determined under a 40× microscope (Orłowski et al. 2016). Prey from the 1 mm sieve was identified to the lowest taxonomic level possible and weighed at the class level. Beetle specimens were identified using the support of entomologists at the Natural History Museum utilising samples of their exoskeleton (i.e., elytra). Small mammal bone samples (i.e., teeth) were identified using a photographic guide (Ramsey and Crawley 2024). Amphibians and fish were excluded as they were fully digested (Vrezec 2009).

### 2.3 Statistical Analysis

All statistical analyses were performed in JAMOVI version 2.3.21 (JAMOVI 2024). We reduced the sample size for the supplementary prey analyses to only include pellets (*N* = 15) where the presence and absence of supplementary prey could be determined with confidence, that is, clear signs of chicken remains. For the pellet morphology analysis, we used the full data set containing samples from 29 pellets.

For statistical comparisons of pellet length (cm) and width (cm), we compared means between both wild and captive groups using One‐way Analysis of Variance (ANOVA) tests due to normal distributions of the response variable residuals using a series of Shapiro–Wilk tests (length: W = 0.954, *p* = 0.235; width: W = 0.970, *p* = 0.561). For the ANOVAs, we measured effect sizes using the η^2^ (eta‐squared) metric. To compare mean pellet weight (g) between stork groups, we used a Kruskal Wallis test and reported the effect size using the ε^2^ metric (Shapiro–Wilk: W = 0.790, *p* < 0.001). For these tests, pellet length (cm), width (cm) and weight (g) were fitted as response variables, with stork “group” fitted as the single explanatory variable.

To quantify foraged prey, only specimens that weighed ≥ 0.01 g were included in the statistical analysis. For supplementary prey, the weight of the foraged prey was subtracted from the overall pellet weight. Similar to McKnight and Najab (2010), we compared differences in proportional supplementary prey in the diet of both groups by conducting a Mann–Whitney U test and fitted “proportional supplementary prey (%)” within each pellet as the response variable and “group” as the explanatory variable (Shapiro–Wilk: W = 0.749, *p* < 0.0001). Effect size from the Mann–Whitney *U* test was computed using the *r* metric.

## 3 Results

**FIGURE 1:**
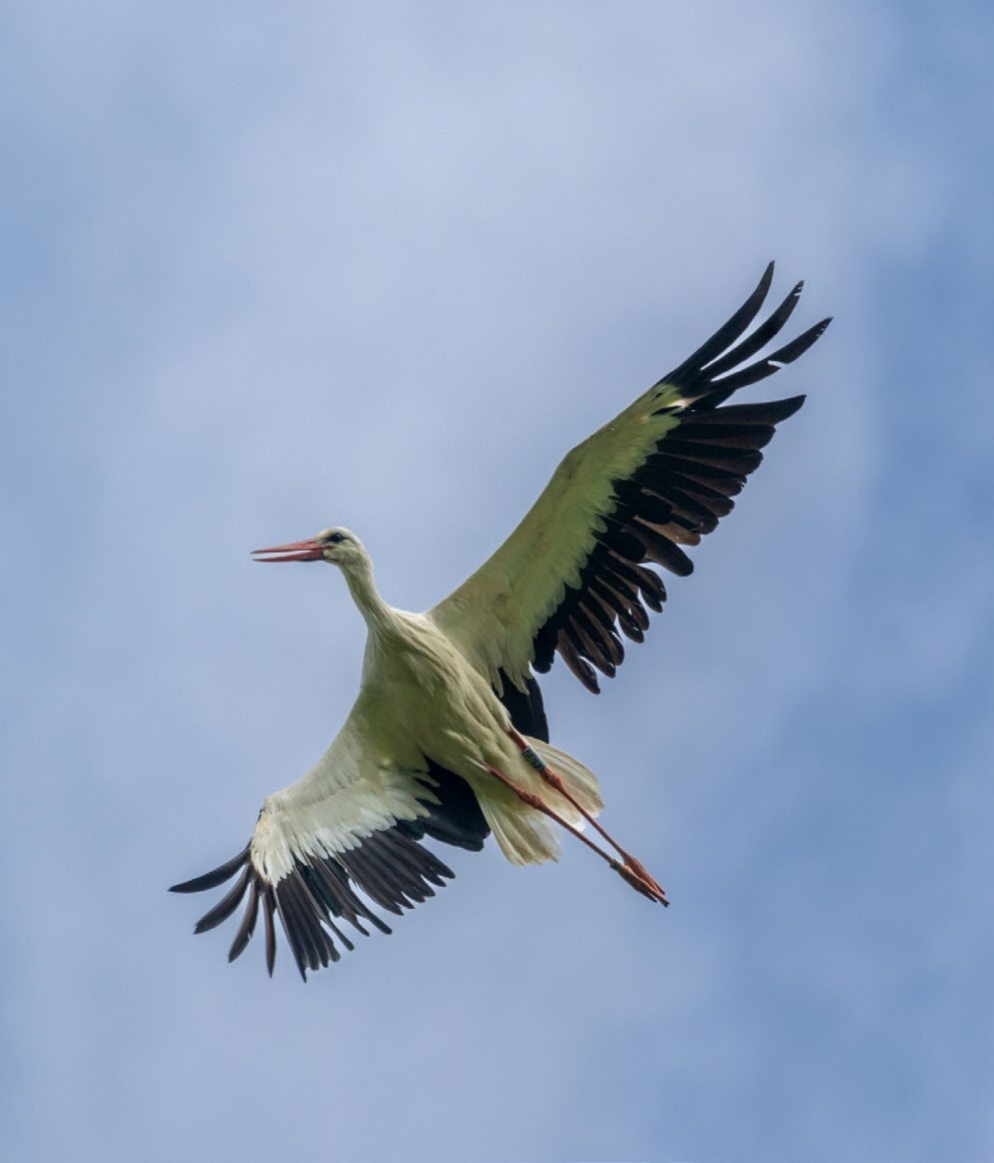
A white stork ( *Ciconia ciconia* ) from the reintroduction programme at Knepp Estate in southern England. Oldham, D. (2023). Email to Şeniz Mustafa, 20 November 2023.

Between June and September 2023, we collected and analysed 29 stork (Figure 1) pellets across the southern block of Knepp Estate. Of these, 10 (34.5%) were from the captive group, and 19 (65.5%) were from the wild group. The captive group enclosure contained 33 individual storks. The wild group pellets were collected around five nests that were occupied by five adult breeding pairs and 12 chicks, 11 of which fledged successfully.

### 3.1 Pellet Morphology Between Groups

Mean pellet length was 5.8 ± 1.6 cm (±SD) for the captive group and 5.3 ± 1.4 cm for the wild group. By width, pellets averaged 3.7 ± 0.7 cm and 3.2 ± 0.7 cm for captive and wild group storks, respectively. Captive group pellets weighed on average 5.2 ± 2.1 g, whereas the wild group pellet mean weight was 12.7 ± 9.2 g. There was no significant difference in pellet length between the captive and wild group birds (F1,27 = 0.711, *p* = 0.407). The effect size (η^2^ = 0.026) suggests that the stork group explained approximately 2.6% of the variance in pellet length, indicating a small effect. The stork group had a medium‐large effect on mean pellet width (η^2^ = 0.099); however, this difference between groups was non‐significant (F1,27 = 2.987, *p* = 0.095). However, the stork group had a large effect on mean pellet weight (ε^2^ = 0.229) with those from the wild group being significantly heavier than captive group pellets (H = 7.208, df = 1, *p* = 0.007).

### 3.2 Foraged vs. Supplementary Food Between Groups

**FIGURE 2:**
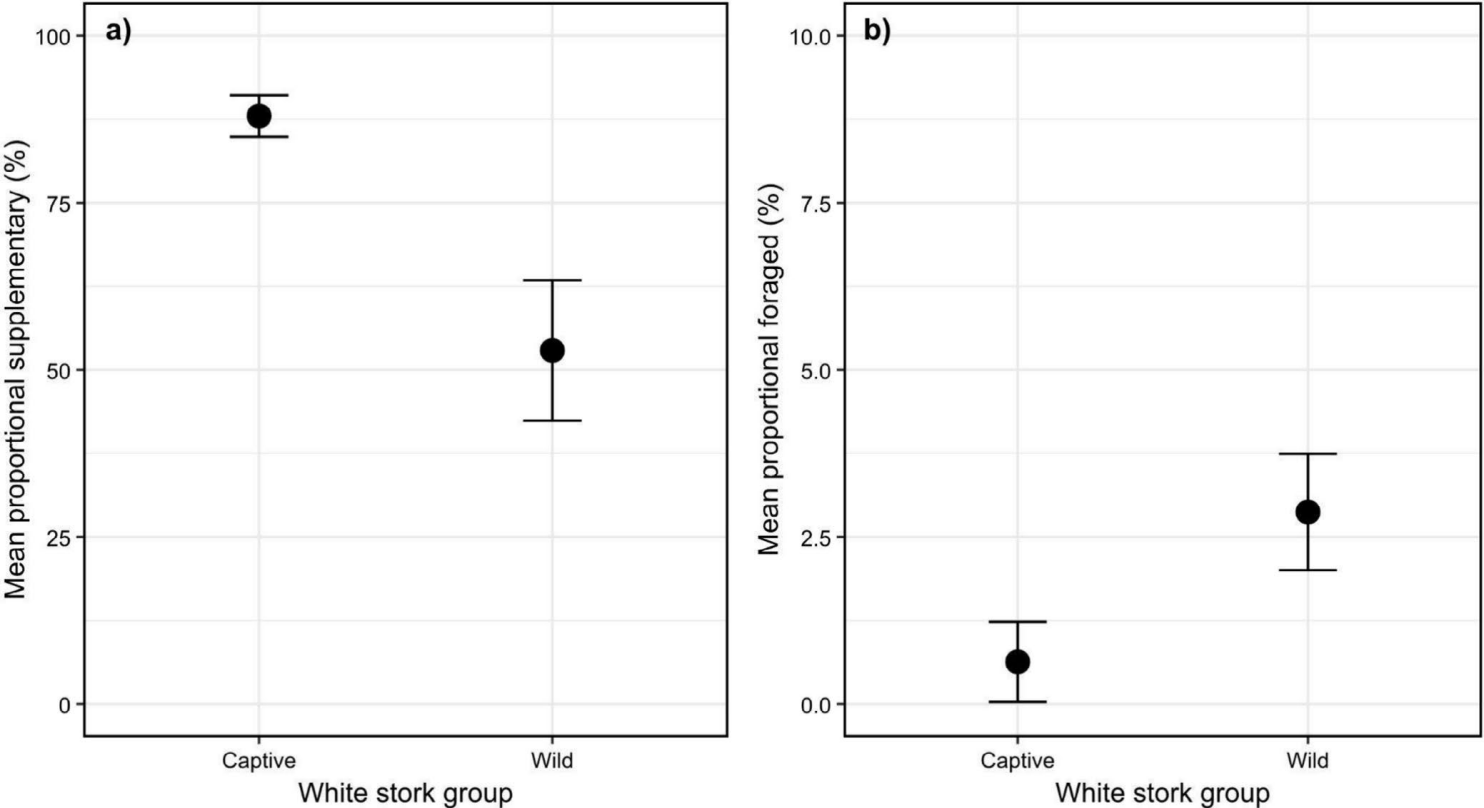
Comparisons of the mean proportional weight (±SE) of (a) supplementary prey and (b) foraged prey within 29 white stork ( *Ciconia ciconia* ) pellets from the reintroduction programme at Knepp Estate in southern England. Proportional comparisons were made between 10 captive group pellets, that is, from individuals bound by a closed enclosure, and 19 wild group pellets, that is, free‐flying individuals. An outlier (80.75% proportional forged prey) from the wild group was removed from graph (a) for visualisation purposes. Note differences in scale.

All 10 pellets from the captive group contained supplementary prey, that is, chicken and sprat, compared to only 73.3% (*N* = 11) of pellets from the wild group. By dietary composition, supplementary prey comprised an average of 88.4% ± 26.1% of captive group pellets and 52.9% ± 36.3% of wild group pellets (Figure 2a). Proportional supplementary prey was significantly higher for the captive group when compared to the wild group (W = 114, *p* = 0.032), with the stork group having a large negative effect on proportional supplementary prey in the stork diet (*r* = −0.397) (Figure 2a).

Contrastingly, foraged prey (Table A1; Figure A1) comprised an average of 0.6% ± 1.5% of captive group pellets and 2.9% ± 3.3% of wild group pellets (Figure 2b). Within wild group pellets, Annelida and Arthropoda were the most frequent phyla. Mammalian (class Mammalia) prey comprised the most weight by class (see Table 1); however, Insecta was the most frequent, and Coleoptera was the most common order (see Table 2). By insect family, Carabidae was the most frequent family across all pellets (Table 3).

**TABLE 1:** The average (±SD) weight (g) of different classes (> 0.01 g) present within 29 white stork ( *Ciconia ciconia* ) pellets sourced from the reintroduction initiative at Knepp Estate in southern England. Proportional between‐family comparisons for 10 captive group pellets, that is, from individuals bound by a closed enclosure, and 19 wild group pellets, that is, free‐ranging individuals, were presented.

Class	Captive group (g ± SD)	%	Wild group (g ± SD)	%
Gastropoda	0.00	0.00	0.01 ± 0.06	0.11
Insecta	0.03 ± 0.10	0.58	0.26 ± 0.40	2.06
Mammalia	0.00	0.00	0.61 ± 1.80	4.75

**TABLE 2:** Dietary composition of 29 white stork ( *Ciconia ciconia* ) pellets from the reintroduction initiative at Knepp Estate in southern England. Proportional comparisons between 10 captive group pellets, that is, from individuals bound by a closed enclosure, and 19 wild group pellets, that is, free‐flying individuals, with prey identified across various taxonomic levels including: Phylum, class and order.

Phylum	Class	Order	Captive (%)	Wild (%)
Annelida			10.00	73.68
	Clitellata		10.00	73.68
Arthropoda			50.00	89.47
	Arachnida		—	5.26
	Insecta		50.00	89.47
		Coleoptera	30.00	89.47
		Diptera	—	47.37
		Hymenoptera	10.00	10.53
Chordata			10.00	26.32
	Mammalia		10.00	26.32
Mollusca			—	10.53
	Gastropoda		—	10.53

**TABLE 3:** Insect families present within 29 White Stork ( *Ciconia ciconia* ) pellets as part of a reintroduction initiative at Knepp Estate in southern England. Proportional between‐family comparisons for 10 captive group pellets, that is, from individuals bound by a closed enclosure, and 19 wild group pellets, that is, free‐ranging individuals, were presented.

Family	Captive group (%)	Number of individuals (±SD; captive)	Wild group (%)	Number of individuals (±SD; wild)
Carabidae	30.00	1.90 (5.32)	78.95	6.89 (10.22)
Elateridae	—	—	36.84	2.11 (3.46)
Silphidae	—	—	26.32	0.47 (0.84)
Staphylinidae	—	—	26.32	0.32 (0.58)
Scarabaeidae	—	—	15.79	0.16 (0.37)
Tenebrionidae	—	—	10.53	1.37 (5.73)
Calliphoridae	—	—	10.53	0.68 (2.54)
Byrrhidae	—	—	10.53	0.26 (0.93)
Dytiscidae	—	—	10.53	0.16 (0.37)
Curculionidae	—	—	5.26	0.11 (0.46)
Formicidae	10.00	0.10 (0.32)	—	—

By weight, supplementary prey comprised 90.9% of the biomass of captive group pellets and 53.4% of the biomass in wild group pellets. A total of 60% of the captive group pellets contained foraged prey compared to 94.7% of pellets from the wild group. Approximately 7% of wild group pellet biomass comprised foraged prey with a mean of 0.9 ± 11.9 g. Foraged prey totalled only 0.6% of the captive group pellet biomass and averaged 0.03 ± 0.1 g.

## 4 Discussion

This study represents the first quantitative dietary assessment of reintroduced storks in southern England. As expected, the wild group pellets were more diverse in prey composition compared to the captive group. Our results clearly demonstrated that wild group individuals successfully forage for themselves. Using diet as a proxy for reintroduction success (Muposhi et al. 2014), we showed that soft‐release techniques can support the reintroduction of individuals to their release sites.

Wild group individuals consumed a range of different taxa, predominantly insects, earthworms, small mammals, and aquatic gastropods such as the Great Ramshorn snail (*Planorbarius corneus*). These prey species are consistent with previous research on stork breeding season diets in Western Europe (Carrascal et al. 1990; Orłowski et al. 2016; Tsachalidis and Goutner 2002), indicating successful acclimatisation following the conservation translocation. However, we did not find evidence of reptiles (Tryjanowski et al. 2018) or birds (Orłowski et al. 2018) in the diets of either group, which may reflect an abundance of and preference for invertebrate prey throughout the release site (Massemin‐Challet et al. 2006; Orłowski et al. 2018; Terraube and Arroyo 2011). Weather conditions and seasonal influx of organic material may explain why we observed a large proportion of earthworm prey in wild group pellets (Chard et al. 2018).

Knepp Estate is a rewilding site where active management of habitats with machinery is not permitted, which is often linked to invertebrate population declines (Golawski and Kasprzykowski 2021). The rewilding approach at Knepp Estate has increased invertebrate diversity throughout the site (Godbehere 2019), which was reflected in our dietary data for the wild group. Furthermore, the presence of mammalian herbivores on‐site provides microhabitats for faeces‐dependent detritivorous insect prey such as Scarabaeidae beetles (Pestka et al. 2023). Key indicator taxa such as beetles were the most abundant invertebrate prey recorded in the wild group pellets, indicating high‐quality habitat across the release site (Rainio and Niemelä 2003; Orłowski et al. 2019). Despite this, storks from both groups foraged across multiple trophic groups, as would be expected from opportunistic feeders (Chenchouni et al. 2015), further supporting high habitat quality both inside and outside of the release enclosure.

During the breeding season, adult storks remain close to the nest (Hilgartner et al. 2014) and are dependent on the habitats surrounding nest site locations. A baseline habitat survey of Knepp from 2005 found the land to be predominantly grassland (English Nature 2005). However, more recent data report a decline in grassland areas due to secondary succession, with a more even balance of grassland and scrubland habitats (Bühne et al. 2022). Surveys from 2005 and 2007 (English Nature 2005; Greenway 2007) detail wetlands/open water at 2% land coverage, with storks being observed utilizing tributaries of the River Adur located near their nests. Wetlands are important foraging habitats for white storks due to the abundance and biodiversity of prey species (Pestka et al. 2023). Although this study found limited aquatic prey, we could not determine the presence of fish and amphibians. Nevertheless, ensuring suitable wetland availability within the proximity of nest site locations will likely benefit breeding storks as their population expands.

Some studies report on stork prey preferences (Antczak et al. 2002; Carrascal et al. 1990; Kwieciński et al. 2015), such as Orłowski et al. (2019) suggesting storks primarily feed upon invertebrates as small and low‐energy prey items that can be opportunistically exploited. Other studies specify further detailing earthworms (Carrascal et al. 1990) or insects, particularly orthopterans and coleopterans, as an important food source (Tsachalidis and Goutner 2002). In this study, insects were the most common prey, followed by earthworms. Other research finds that storks prefer small mammals, predominantly voles (*Microtus* spp.) (Orłowski et al. 2016; Tobolka et al. 2012; Tryjanowski et al. 2018) and occasionally moles ( *Talpa europaea* ) (Vrezec 2009). A study on storks in Poland found that voles occurred in 87% of pellets and constituted 43% of the pellet biomass (Antczak et al. 2002). Similarly, Kwieciński et al. (2015) suggested that storks may prefer small mammal prey due to high calorific and protein contents, with individuals avoiding insects and not consuming earthworms. Although Pinowski et al. (1991) also found that voles, as opposed to insects, comprised the most important prey for storks, we found limited evidence of small mammal prey in this study, despite remains of a field vole ( *Microtus agrestis* ). The absence of small mammals in the diets of storks at Knepp Estate may be due to their partial reliance on supplementary prey, whilst insects remained their favoured secondary food source, or due to the limited temporal time span of our survey period. For pellets that contained mammalian remains, supplementary prey was absent, indicating a dietary shift towards small mammals.

There was evidence of the consumption of vegetation; however, this may be attributed to accidental consumption whilst foraging (Antczak et al. 2002; Milchev et al. 2013). Diptera species, namely *Calliphora vomitoria* , were commonly found within pellets but in the form of puparia and casters. However, it is possible they were sourced from infested foraged or supplementary prey. Stones were observed in three pellets, which could act as gastroliths to aid digestion (Rosin and Kwieciñski 2011; Duffin 2012). Due to the limited size of our study site and short fieldwork season, our statistical approach was limited by the sample size of our data set. Subsequently, we could not account for nest‐level variation in the diet of wild group storks using more robust statistical approaches such as linear mixed modelling. Future studies should collect additional pellet data across longer timescales and employ more robust statistical methods to improve our understanding of how the stork diet responds to conservation reintroductions. We were also unable to control for fragmented pellets and pellet condition (i.e., freshness) in the field, which may influence our morphology results and should be considered when inferring from our findings.

### 4.1 Implications for Conservation Reintroductions

As expected, the greatest proportion of the wild group's diet by weight comprised supplementary prey. Reliance on supplementary prey can be explained by optimal foraging theory (OFT), which states that animals should make choices that maximize their net energy gain whilst foraging (Sjöholm 2024; Watanabe et al. 2014) by selecting prey items high in energy and nutrients (Bartumeus and Catalan 2009; Støstad et al. 2017). This is particularly emphasized during the breeding season when energy demands are highest (Dreyer 2024), with individuals minimizing energy expenditure through the distance traveled between nesting and foraging sites (Bartumeus and Catalan 2009; Nilsson et al. 2020; Sky et al. 2024). This is known as central place foraging (CPF) (Alerstam et al. 2019; Tremblay et al. 2022) and is demonstrated by the close proximity of stork nests to the feeding site. Supplementary feeding sites act as stable and reliable food sources (Bialas et al. 2019), allowing more time for offspring care and protection (Galbraith et al. 2015). This has been demonstrated in Red Kites ( *Milvus milvus* ), which found that individuals occur more commonly in residential areas because of household provisioning of supplementary or anthropogenic food (Orros et al. 2015).

### 4.2 Conclusions and Future Research

Our study was restricted temporally to a single breeding season, and future research should focus on analysing dietary trends over multiple years. Additionally, more advanced techniques, such as stable isotope analyses (Inger and Bearhop 2008; Jones et al. 2024), could further enhance our understanding of how reintroduced individuals adapt to new environments, providing crucial insights into the success of conservation translocations. Given this, our study represents southern England's first quantitative dietary assessment for reintroduced storks. We found that the wild group storks had an increased dietary diversity and a similar diet to conspecifics elsewhere in their geographical range, indicating successful acclimatisation to the release site. As a result of the rewilding initiative, we discovered that the habitats at Knepp Estate are suitable for stork foraging. However, we recommend increased access to wetlands and a greater mosaic of grass lengths could provide storks with better access to small mammals once supplementary feeding has ceased. Dietary data presented in this study suggest that storks can survive without supplementary provision and establish a self‐sustained breeding population in the future.

## Author Contributions

**Şeniz Mustafa:** conceptualization (lead), data curation (lead), formal analysis (lead), investigation (lead), methodology (lead), project administration (lead), validation (equal), visualization (equal), writing – original draft (lead), writing – review and editing (equal). **Connor T. Panter:** visualization (equal), writing – review and editing (equal). **Laura Vaughan‐Hirsch:** conceptualization (supporting), resources (lead). **Rachel L. White:** conceptualization (equal), methodology (supporting), validation (equal), writing – review and editing (supporting). **Anja Rott:** conceptualization (equal), methodology (equal), validation (equal), writing – review and editing (supporting).

## Conflicts of Interest

The authors declare no conflicts of interest.

## Supporting information


Appendix S1.

## Data Availability

The authors confirm that all data that supports the findings of this study are fully available through Figshare without restriction (https://figshare.com/s/8e40330f6b89162fdabe. DOI: 10.6084/m9.figshare.28287248).
