# Supplementary material for: Reintroduced White Storks ( Ciconia ciconia ) Have Similar Diets to Their Wild Conspecifics
Source: Ecol Evol. 2025 Apr 11;15(4):e71278. doi: 10.1002/ece3.71278 (PMC11986844; doi:10.1002/ece3.71278)
Supplement: Supplementary file 1 — Appendix S1. [file ECE3-15-e71278-s001.docx]

**APPENDICES**

Table A1. Species composition of taxa found within 19 free-ranging White Stork (*Ciconia ciconia*) pellets as part of a reintroduction programme at Knepp Estate in southeast England.

| Order | Family | Species |
| --- | --- | --- |
| Basommatophora | Planorbidae | *Planorbarius corneus* |
| Coleoptera | Carabidae | *Carabus violaceus* |
|  |  | *Harpalus affinis* |
|  |  | *Poecilus cupreus* |
|  |  | *Pterostichus melanarius* |
|  |  | *Pterostichus niger* |
|  | Dytiscidae | *Agabus bipustulatus* |
|  |  | *Dytiscus marginalis* |
|  | Elateridae | *Agriotes obscurus* |
|  |  | *Agriotes linneatus* |
|  |  | *Agriotes sputator* |
|  |  | *Hemicrepidius hirtus* |
|  | Scarabidae | *Hoplia philanthus* |
|  | Silphidae | *Phosphuga atrata* |
|  |  | *Silpha tristis* |
| Diptera | Calliphoridae | *Calliphora vomitoria* |
| Rodentina | Cricetidae | *Microtus agrestis* |


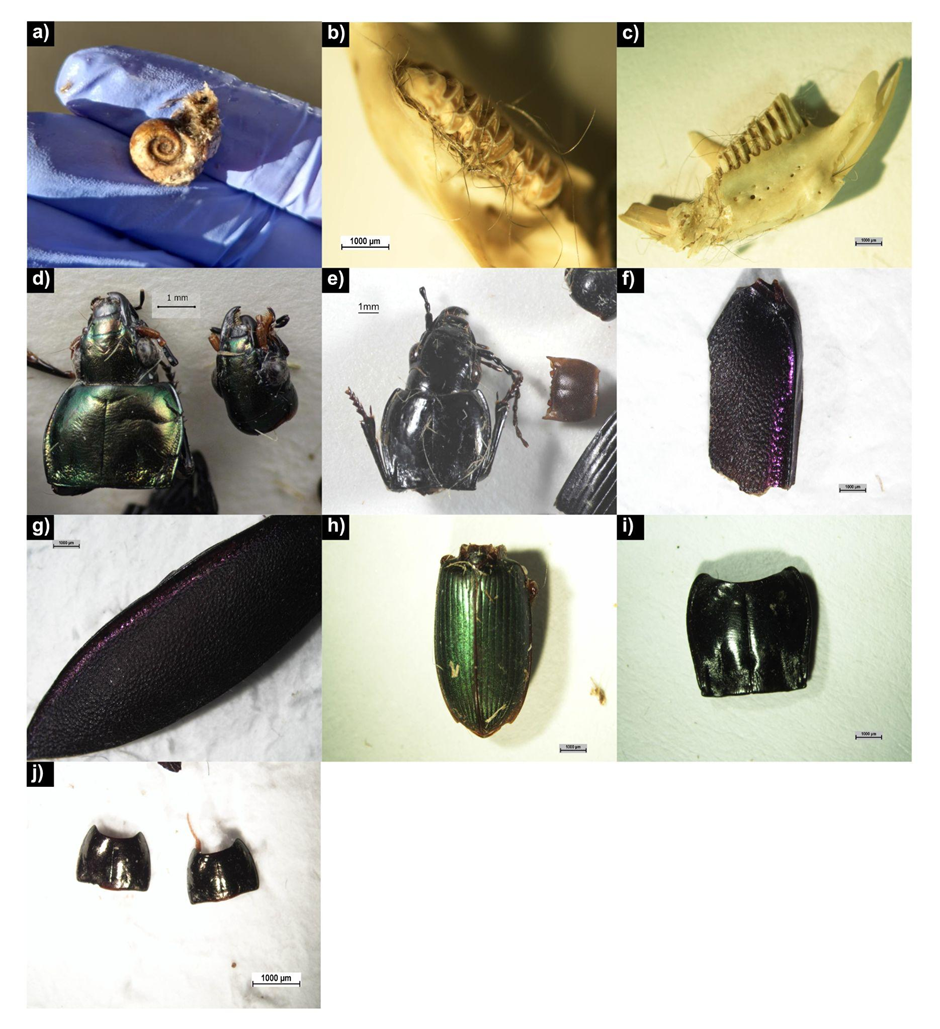
Figure A1. A sample of specimens found within free-flying white storks (*Ciconia ciconia*) pellets as part of a reintroduction programme at Knepp Estate in southeast England (a) *Planorbarius corneus* (b-c) *Microtus agrestis* (d) *Poecilus cupreus* (e) *Pterostichus melanarius* (f-g) *Carabus violaceus* (h) *Harpalus affinis* (i) *Pterostichus niger* (j) *Amara spp.*
